# A Few Extracts from “Fox and Harris” in Contrast

**Published:** 1902-01

**Authors:** Henry C. Spencer

**Affiliations:** ’97, Newton, Mass.


### A FEW EXTRACTS FROM "FOX AND HARRIS" IN CONTRAST.[1]

BY HENRY C. SPENCER, D.M.D., '97, NEWTON, MASS.

In the few minutes which are allotted to me, I wish to read to you a few promiscuous extracts from one of the first text-books published in the interest of the dental profession, believing that it will have some interest especially to the younger members of the profession who have, perhaps, found difficulty in keeping up with the dental literature even of the present day. It would be interesting to know the limitations of the profession of the future. It is interesting to know the difficulties under which the practitioners labored in the past, that we may better appreciate the advantages which we of the present day possess, and which advantages we must strive to increase in order that the dental profession shall be one of constant progression. When we think of the practitioners of the early part of the last century, we are sometimes apt to liken them to the traditional blacksmith, and as possessing about the same delicacy of touch as he would be likely to possess. We are apt to think of them as being unprofessional and unrefined; and yet the dental literature of that period was as strong in the true professional spirit as it is to-day, as will be evident from the following from one of the first text-books published in the interest of the profession in 1840.

"The treatment of the diseases of the teeth, and the replacement of their loss with artificial substitutes, do not comprise all the duties of the surgeon-dentist. The treatment of the various afflictions of the gums, alveolar process, and their contiguous parts, as well as the management of second dentition when affected in a faulty and improper manner, and the correction of the irregularity in the arrangement of these organs, all come legitimately within his province. He, therefore, who would be a successful practitioner, should not only be skilled in the various mechanical manipulations which belong to it, but he should also have a knowledge of anatomy, physiology, pathology, and the therapeutical indications of disease generally. Without this knowledge no one should take upon himself the responsibility of practising the profession, and to obtain it requires much time and close and persevering application. Neither

---

[1] Read before the Harvard Dental Alumni Association, June 24, 1901.

mechanical ability nor the highest medical attainments, nor both combined, without a thorough knowledge of the diseases of the dental apparatus and their treatment, can make a successful practitioner of this branch of the healing art.

"That any one, therefore, should be guilty of the folly of committing the treatment of diseases of organs so valuable as the teeth to any individual totally destitute of all qualifications, and having no other claim to skill in their management than the mere assumption of the name of dentist, is almost incredible. Such inconsistency might seem paradoxical, if it were not constantly observed in individuals moving in the most learned and polished walks of society and manifesting in most matters great prudence, shrewdness, and judgment. But this is not so culpable in others when medical men, eminent for erudition and skill in their profession, have been known to employ and recommend men of this description. Thus encouraged, they have multiplied with astonishing rapidity; and if it were not that some men of education, talent, and ingenuity are engaged in this field of practice, they would long before now have destroyed all confidence in the alleviatory resources of this profession. But, thanks to the efforts and unwearied labor of such men, notwithstanding the increase of empirics, the progress of the science and art of dental surgery has been rapid. It has outstripped the most ardent flights of imagination, and has already attained a degree of excellence which a few years ago was supposed impossible."

It will thus be seen that the most reputable practitioners in the early part of the last century, although possessed of what we would now consider a very limited knowledge of dentistry, were nevertheless keenly alive to the advantages to be gained by the most thorough knowledge then attainable, and were extremely jealous of those who practised without the best education to be obtained at that period.

Up to within fifty years ago extracting was the principal method of relieving a troublesome tooth; and this sometimes leads us to believe that the early practitioners were not aware of the importance of retaining the natural conditions as they should be, especially in regard to the temporary teeth. Yet from Fox and Harris, published as early as 1840, I take the following:

"Sound teeth are as desirable and just as necessary to the comfort and health of a child as they are to an adult, and, therefore, they should not be permitted from neglect to decay, and the temporary teeth require as much care as do the permanent ones, and they should never be extracted except for the relief of pain,—that cannot be removed by any other means,—or the cure of an abscess, or to make room for a permanent tooth. The popular opinion that, inasmuch as these teeth are to be replaced with others, it is of little importance whether they remain in the mouth until they are removed by nature to make room for their successors or are lost a year or two earlier is erroneous, and has been productive of much injury."

So it seems that while the practitioner of this early period held the same views in regard to keeping the teeth as nearly as possible as nature intended, as we do to-day, others seem hard to understand when viewed from this age of progression; but when we realize they had no knowledge of bacteriology, which has revolutionized so many theories in the past few years, some of their views are not to be wondered at.

Fox and Harris, for instance, in speaking of caries, say, " Caries, or, as it is commonly called, decay, is the disease with which the teeth are most frequently affected. At first it has its origin in the bony part of the crown of the tooth, the structure of which is gradually destroyed, and the disease proceeds until the whole crown of the tooth, both the enamel and the bone, is entirely removed. The enamel, though *less* frequently, is nevertheless sometimes first attacked. On the mastication of hard substances, pieces of the enamel are broken off on account of the texture of the bony part being destroyed by the caries which had previously gone on internally. If a sound tooth that has been recently extracted be broken, the membrane will be found to be firmly attached to the bone of the tooth forming the inner cavity. But when this membrane becomes inflamed, it separates from the bone, and the death of the tooth is the consequence.

" That this is the proximate cause of caries appears to be highly probable, by remarking that a caries of other bones is caused by a separation of those membranes which cover them and which are attached to them. Thus a separation of the periosteum will cause a death of part of the tibia or that part of the pericranium, a caries of some part of the bones of the head. This opinion is also confirmed by comparing the symptoms which accompany inflammation in a bone with those which are occasionally felt by persons in their teeth previous to any appearance of caries. When the inflammatory symptoms subside, the pain in the teeth goes off; but as inflammation may have caused the death of some part of one or more teeth, the decomposition of the internal part of the tooth goes on until the enamel is broken away and caries is discovered. I could mention many cases in corroboration of this statement, and produce several examples of teeth with the decay extending through the internal part while the enamel remained perfectly sound.

"That teeth acquire a disposition to decay through some want of healthy action during their formation seems to be proved by the common observation that they become decayed in pairs. That is, those teeth which are formed at the same time, being in a similar state of imperfection, have not the power to resist the causes of disease, and, therefore, nearly about the same period they exhibit signs of decay, while those teeth which have been formed at another time, when a more healthy action has existed, have remained perfectly sound to the end of life.

"TREATMENT OF CARIES.

"When the decay is situated on that side of a tooth which is in opposition to another, so that persons say the decay is between two teeth, it is always difficult, and frequently impossible, to retain the stopping, in which case great inconveniences arise from the food lodging in those cavities, whence it is not easily removed. Great benefit will here be derived from passing a file between the teeth, in which operation the opening should be so much enlarged as to allow a quill tooth-pick to be used with ease.

"When the decay has considerably advanced, a small round or half-round file may be used; and it should be carried into the mouth in an oblique direction, so as to preserve as much as possible of the front part of the tooth. If the tooth is very sensitive, file a little at a time until the decay has been nearly or quite eradicated, recommending, during the intervals, the application of spirits of wine to the decayed parts, which tends to harden the caries substance of the tooth and to diminish its sensibility. To many the sensation produced by the action of the file on the teeth is exceedingly disagreeable, and to some quite painful; but the operation should never be suspended on this account."

"AMALGAM.

"The most objectionable and damaging article that has ever been employed for filling a tooth is an amalgam of mercury and silver known by the various names of *lithodeon, mineral cement,* etc. This preparation not only really oxidizes, but also turns the teeth black, and causes them to decay more rapidly than if let alone. It moreover exerts a hurtful influence upon the *alveolo-dental periosteal* tissue, gums, and, in fact, the whole body. A number of marked cases of salivation produced by the use of this amalgam have fallen under the observation of the editor. Yet it has been most extravagantly eulogized by a few unscrupulous empirics during the last six or seven years, and thousands of teeth in Europe and America have been destroyed by it.

"TOOTHACHE, AND TO DESTROY NERVE.

"Attempts have been sometimes made to destroy the nerve with the actual cautery by introducing a red-hot wire; but I have scarcely ever found this plan to be effectual, and, as it always gives great pain, and sometimes produces an increase of inflammation, I think it better never to recommend it. Indeed, all applications to stop toothache are uncertain, and if relief be not at once obtained it is better to extract. It was ascertained by Mr. Spooner, of Montreal, that the nerve of a tooth might always be destroyed in a few hours by the application of a small quantity of $AS_2O_3$. At first the discovery proved to be of great value; but though it was ascertained that the nerve could with certainty be destroyed by it, and the tooth afterwards plugged, alveolar abscess was almost sure to result. The use of it has therefore been almost altogether abandoned by the more skilful and experienced of the profession."

(Preferred raising in socket, or in case of anterior teeth to extract, fill, and replace.)

CROWNING.

Files, etc., about same as now. Then the nerve was destroyed by passing a small iron or steel wire, heated until it was white, suddenly up the canal. The destruction of the nerve in this manner is exceedingly painful, several applications being sometimes necessary, and in some cases producing alarming consequences; but, notwithstanding, is recommended by many of the most reputable practitioners. Since the employment of $AS_2O_3$ for the destruction of nerves in teeth, it has been used to some extent for this purpose; but it is objectionable in that it not only destroys the whole of the lining membrane of the tooth, but it also induces an unhealthy action in the investing membrane, which generally in the course of a few months terminates in alveolar abscess, thereby rendering the root unfit for the support of a crown as well as obnoxious to the surrounding parts. It is important, therefore, that as much of the vitality of the root as possible should be preserved. The author recommends as the *best* method of destroying the nerve to take a small iron or silver wire with the point brought to an edge and left rough. The manner of performing the operation is to introduce the instrument into the canal about one-half or five-eighths of an inch, giving it at the same time a quick rotary motion, which will extirpate the nerve as far up as the broach extended, leaving the remaining portion and lining membrane to supply the inner walls of the fang with vitality and nutriment, which they will oftentimes do for years. The patient should be instructed beforehand that the operation will be attended with considerable pain, but that it will be of but a *few seconds' duration.*

